# Worldviews, values and perspectives towards the future of the livestock sector

**DOI:** 10.1007/s10460-023-10469-9

**Published:** 2023-06-07

**Authors:** Kirsty Joanna Blair, Dominic Moran, Peter Alexander

**Affiliations:** 1 Global Academy of Agriculture and Food Security, The Royal (Dick) School of Veterinary Studies, University of Edinburgh, Easter Bush Campus, Midlothian, EH25 9RG UK; 2 School of Geosciences, University of Edinburgh, Drummond Street, EH25 9RG Edinburgh, UK

**Keywords:** Sustainability, Livestock, Worldviews, Values

## Abstract

The livestock sector is under increasing pressure to respond to numerous sustainability and health challenges related to the production and consumption of livestock products. However, political and market barriers and conflicting worldviews and values across the environmental, socio-economic and political domains have led to considerable sector inertia, and government inaction. The processes that lead to the formulation of perspectives in this space, and that shape action (or inaction), are currently under-researched. This paper presents results of a mixed methods exploration of the influence of environmental worldviews, values, and demographic factors on perspectives towards the future of the livestock sector. The approach combines survey and interview data derived from a sample of livestock representatives (N = 307). Respondents with higher pro-environmental, ecocentric and relational worldviews and values favour more behaviour-oriented solutions. Those with lower pro-environmental and higher techno-centric worldviews and values favour technological solutions to improve the efficiency of production and to enable continued patterns of meat consumption. Demographic variation and qualitative data emphasise the need to recognise cultural and geographic nuance in narratives. This study improves our understanding of the processes that lead to the formulation of perspectives, enabling the development of more holistic solutions that acknowledge all voices in an increasingly polarised debate. Adopting more pluralistic, relational methodologies will therefore be paramount in developing solutions for sustainable livestock futures.

## Introduction

Modern-day food systems have been described as being both miraculous and disastrous (NFS, 2021). We have defied Malthusian mass famine predictions, producing enough calories to feed 7.9 billion people. However, uneven distribution of food resources has resulted in inequalities between countries, within countries and even within households resulting in problems at both extremes. Consumption of meat is expected to increase by 75–145% by 2050 (Godfray et al. 2018), with a large proportion of the increase expected in developing countries as a result of population rise, increasing affluence and shifts in dietary preference. However, developed countries currently consume significantly higher proportion of meat per capita (OECD, 2021). Although growth in meat consumption is slowing in developed countries, with some suggesting a shift in preferences related to income (Vranken et al. 2014; Gallet 2010). The uneven distribution and consumption of animal source foods is problematic. Nearly 800 million people do not have access to animal source foods, leading to micronutrient deficiencies (Adesogan et al. 2020). Meanwhile, overconsumption of red and processed meat in developed countries has resulted in increased rates of obesity and non-communicable diseases (Wellesley et al. 2015). Food insecurity in developing countries is also being threatened by land use change with increased demand for animal feed (Makkar 2018).

Furthermore, intensification of livestock systems can accelerate disease transmission between animals due to increased population size and proximity, which often leads to increased antimicrobial use to tackle disease and promote growth, and this has been associated with increased antimicrobial resistance (Jones et al. 2013). Agricultural intensification and environmental change have also been associated with increased risk of zoonotic disease (Jones et al. 2013). In addition to these health challenges, the Food and Agriculture Organization of the United Nations (FAO, 2013) estimated that the livestock sector produces 7.1 gigatonnes CO_2_e^1^ annually, representing 14.5% of human-induced greenhouse gas emissions (GHGs). Beef and cattle milk are the largest contributors (41% and 20% respectively), followed by pig meat (9%) and poultry meat and eggs (8%). According to the Climate and Clean Air Coalition 2030 Strategy, mitigating short-lived climate pollutants like methane will be vital to keep warming below 1.5 °C and to avoid crossing further devastating and irreversible tipping points (CCAC, 2020). Moreover, meat production and crops for feed account for almost one-third of global deforestation and the associated GHGs (European Commission, 2013). The production of meat is also extremely resource-intensive in terms of land, water and energy use (Wellesley et al. 2015). Additionally, the production and use of industrial fertilisers, like ammonia, not only release carbon, but can also seep into watercourses. Nitrogen run-off from fertilisers and slurry can result in eutrophication, raised pH levels and processes that create hypoxic or anoxic zones in freshwater and the sea, negatively impacting aquatic ecosystems (NFS, 2021).

Overall, plant-based proteins have a much smaller environmental burden than beef, accounting for GHG emissions, land use, terrestrial acidification and eutrophication (Poore and Nemecek 2018). For example, the mean GHG emissions for beef (beef herd) are 99.5 kg CO_2_e compared to just 3.2 kg CO_2_e for tofu. Though the environmental burden of producing the same goods have also been shown to be highly variable. Monogastric or soy/grain-fed livestock animals can also be inefficient converters of calories; 36% of calories produced globally are fed to these animals, yet just 12% of those feed calories ultimately contribute to the human diet through animal source foods (Cassidy et al. 2013; Alexander et al. 2017) found that livestock production i.e. the conversion of feed and grass to animal products, resulted in the highest losses in terms of dry/wet mass, energy and protein, when compared to agricultural production, handling, storage and transportation, processing, consumer waste and over-consumption.

These sustainability challenges have led to several international and UK-based inquiries calling for dramatic changes to current patterns of livestock consumption and production (e.g. IPCC, 2019; Willet et al., 2019; CCC, 2020; NFS, 2021). However, despite clear recommendations, institutional and biological complexity, a lack of communication and an overreliance on the market have led to a cycle of sector inertia, and government inaction (Wellesley et al. 2015; Garnett et al., 2015). Industry lobbyists have also been accused of trying to “confuse and delay regulation” (Christen 2021; Dunne 2021). However, confusing and often misleading rhetoric can be detected on both sides of the debate.

As with other sustainability challenges, there is a range of complex, divergent and conflicting worldviews and values within the livestock sector across the environmental, socio-economic, and political domains. Worldviews and values are complex and multifaceted concepts that have been explored extensively in the academic literature. The term ‘worldview’ is often used to describe the way that individuals and societies make sense of the world and their place in it. This encompasses, for example, the social constructs that allow us to understand and derive meaning from the world around us, such as what can be known, how knowledge is generated, the constituents of reality and morality, and our ultimate aspirations (Johnson et al. 2011). They encompass our beliefs, assumptions, values, and cultural norms, and can be shaped by factors such as religion, culture, language, and historical context. On the other hand, ‘values’ is a term often used to describe what is important or desirable to us as individuals or as a society, helping us make decisions and prioritise our goals, shaping our beliefs, attitudes, behaviours, and rationalisations (Williams 1968; Rokeach 2008). The relationship between worldviews and values is complex and often intertwined. Our values can be shaped by our worldview, and vice versa (Holmes et al. 2012). It is important to recognise that worldviews and values are not static and can change over time in response to various factors. These include, but are not limited to, cultural and societal transformations, personal experiences, disruptive events, communications, policies, new information and ideas, repeated exposure to different values, and social and political movements (Holmes et al. 2012).

Since the green revolution, the agriculture sector has become increasingly dominated by the productionist worldview, which values productivity, efficiency, and profit over other social and environmental considerations (Bawden, 2006; Page and Witt 2022). This worldview is reinforced by government policies that prioritise economic growth and trade liberalisation, encouraging intensive and industrialised forms of agriculture. The result is an exploitation of natural resources for human gain and a hierarchical relationship between humans and nature, which has led to environmental degradation and social inequality. Bawden (2006) suggests that the productionist worldview also presents significant epistemic impediments to the adoption of more systemic or holistic worldviews that are better suited for dealing with the complexity of sustainability challenges in the agricultural sector. To address these challenges, there is a growing recognition of the need for promoting alternative worldviews and agricultural models that align with the core principles of sustainability, including meeting the needs of present and future generations while promoting social equity, human welfare, and preserving natural resources (Page and Witt 2022). In order to address sustainability challenges in the agro-food and livestock sectors, there has been a growing appreciation of the complex interactions of worldviews and values in influencing transformative change (Bawden, 2006; Cayre et al. 2018; Plumecocq et al. 2018; Laininen 2019; El Bilali 2019; Dumont et al. 2021; Page and Witt 2022).

This study contributes to the existing literature by empirically investigating the relationship between environment worldviews, values, demographics and future livestock perspectives for livestock sector representatives. By recognising the importance of worldviews and values in shaping decision-making and developing a more nuanced understanding of diverse perspectives, there is an opportunity to identify reasons for inertia and promote inclusive and equitable policy solutions to help create a more sustainable future for the livestock sector. The following sections provide an overview of the survey and interview design, data analysis, and results highlighting the significance of environmental worldviews, values, and demographic factors in influencing stakeholder perspectives on livestock futures. The discussion section presents policy insights and implications for the future of the livestock sector. Finally, the conclusion synthesises the study’s findings.

## Materials and methods

### Survey design and analysis

A survey was designed to uncover how environmental worldviews and values influence perspectives towards the future of the livestock sector. The survey targeted respondents involved in the livestock sector in any way including, researchers, students, veterinarians, farmers, producers, processors and others. This ensured broad representation of perspectives and helped to determine how perspectives might differ across the sector.

The survey contained five sections. To understand what constitutes a solution, we must first consider perspectives towards the problem in current livestock systems. Section one therefore explored participant reactions towards different problem statements including, there is ‘Not enough food’, ‘Too much greed’ and ‘Too much inequality’ – see Table 1 for definitions adapted from Garnett (2015). Participants responded to the statements on a five-point Likert scale from strongly disagree to strongly agree.

**Table 1:** Perspectives towards the problem in the livestock sector. (adapted from Garnett 2015)

Problem	Description Summarised/adapted from Garnett (2015, pp.4–8).
Not enough food	There is not enough food to feed the growing population. We need human innovation to increase the efficiency of meat production.
Too much greed	Overconsumption of meat is part of a bigger environmental problem of human greed – the desire to consume more and more. Our consumption patterns are resource intensive, unhealthy and must change.
Too much inequality	There are problems of excess and insufficiency both in the environment (over and under-use of agricultural inputs); and in our bodies – in the form of obesity and malnutrition. The solution is not necessarily to produce more or less but rather to rebalance the system.

**Fig. 1:**
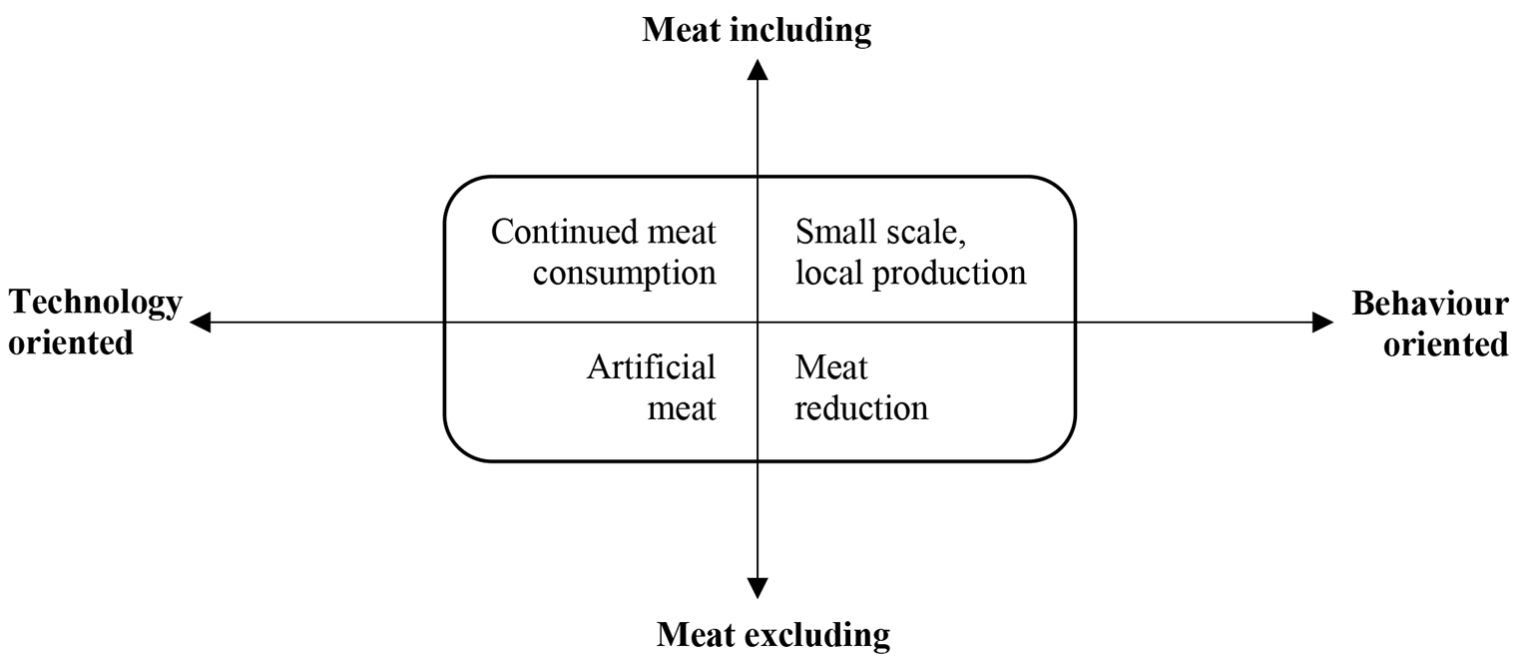
Two-dimensional spectrum, outlining four livestock future scenarios. (adapted from Garnett 2015)

Section two considered participant reactions towards different livestock future scenarios. Perspectives towards the future of the livestock sector could be considered on a spectrum ranging from technology oriented to behaviour oriented, and from meat including to meat excluding, including four possible future scenarios: ‘continued meat consumption’, ‘artificial meat, ‘small scale, local production’, ‘meat reduction’ – see Fig. 1 and Table 2 adapted from Garnett (2015). Participants were asked to select the scenario they would most like and expect to see by 2035.

**Table 2:** Livestock future scenarios. (adapted from Garnett 2015)

Scenario	Description Summarised/adapted from Garnett (2015, pp.9–27).
Continued meat consumption	Meat consumption patterns cannot and should not be moderated. People continue to eat more and more meat globally. There has been significant investment into a wide range of technologies to improve efficiency of meat production and support consumption trends.
Artificial meat	Artificial, lab-grown meat has gone mainstream, including products that mimic quality carcass meat, dairy products and fish. Insect derived foods are available in multiple forms. Other forms of novel protein derived from plants and algae are also widely available.
Small scale, local production	Food production and distribution has shifted towards smaller scale, local systems of production. There is a focus on farming practices that improve animal welfare, are more diverse and environmentally conscious. Livestock are reared on marginal lands rather than prime arable land.
Meat reduction	Meat consumption in developed countries has drastically declined. Developing countries stabilise at a similar level. Diets now largely consist of legumes, meat alternatives, fruits and vegetables. Veganism and vegetarianism are much more common, but most people eat meat once a week.

The third section of the survey considered participant reactions towards twelve policy scenario statements. These statements reflected the four scenarios above with three for each – see Table 3. Participants respond to the statements on a five-point Likert scale from strongly disagree to strongly agree, with a total calculated for each policy scenario.

**Table 3:** Livestock future policy scenario statements

Scenario	Policy scenario statement grouped by scenario
Continued meat consumption	Meat consumption patterns cannot and should not be moderated
	We need to increase the efficiency of meat production - minimising land use and greenhouse gas emissions - to support increased consumption globally
	Alterations to livestock through breeding and genetic modification are needed to improve efficiency of livestock production and to reduce greenhouse gas emissions
Artificial meat	There needs to be investment into developing artificial lab-grown meat as an alternative to conventional meat
	There needs to be investment into developing high protein foods derived from insects as an alternative to conventional meat
	There needs to be investment into developing high protein foods derived from plants and algae as an alternative to conventional meat
Small scale, local production	Food production and distribution need to shift towards smaller scale, local systems of production
	Achieving self-sufficient food systems within countries should be a policy priority
	Livestock solutions should focus on learning from traditional, indigenous and local knowledge
Meat reduction	Meat consumption in developed countries needs to drastically decline
	Governments should intervene, e.g. through livestock taxes or incentives for farmers to shift from animal rearing into other farming or non-farming activities
	Nutritional and environmental education should be a core element in the school curriculum

Note that though these problems, scenarios and policy implications are “improbable in their singleness” (Garnett 2015, p.25), they were chosen to provoke participant reflection on the future of the livestock sector, and the later qualitative responses and interviews designed to uncover nuance.

Section four explored participant’s environmental worldviews and values utilising the revised New Ecological Paradigm scale (NEP) (Dunlap et al. 2000). The NEP conceptualises individual’s environmental worldviews, values, beliefs and attitudes, including fifteen items that measure perspectives towards the reality of limits to growth, anti-anthropocentrism, the fragility of nature’s balance, rejection of exemptionalism and possibility of an eco-crisis (Dunlap et al. 2000). Several studies have shown that high NEP scores are associated with pro-environmental values and ecocentrism (Hunter and Rinner 2004; Kortenkamp and Moore 2001; Gangaas et al. 2015), whereas low NEP scores are associated with technocentrism (Gerhard 2004; Gangaas et al. 2015). Relational values have been shown to be a distinct construct compared to the NEP scale (Klain et al. 2017), therefore are also assessed in the survey using an additional seven statements (adapted from Chan et al. 2016). These statements allow us to understand how people relate with others, with nature and animals, including what it means to lead a ‘good life’, social cohesion, cultural identity, sense of place and core values relating to care, justice, reciprocity and virtue (Chan et al. 2016). Participants responded to the statements on a five-point Likert scale and the sum of the responses was calculated.

Section five explored participant demographics, including occupation, disciplinary background (for researchers/students), years in the sector, livestock sector(s) represented, country, gender and age. Participants were also asked whether they would be willing to participate in a follow-up interview. There was also an option to leave qualitative feedback in the survey for five questions, including reflections on the current problems within livestock systems, what participants would like to see change by 2035, expect to see by 2035, and what participants think should be the research and policy priorities.

Before circulation, the survey was piloted, and questions refined. The final survey was administered online via Qualtrics XM platform and data collected from 17th June-7th July 2021. To incentivise response, £200 worth of gift cards were offered in prize draws. Participants were recruited via a variety of means, including email, social media and forums. In total, 161 diverse organisations, networks and interest groups were contacted. Given that this research involved human participation, ethical procedures were followed in accordance with the University of Edinburgh’s guidelines, including obtaining informed consent, protecting anonymity and secure data handling. Ethics approval was granted in May 2021.

Statistical analyses were conducted using Stata/SE 16.1. A minimum sample size (n = 114) and power were calculated via G*Power Version 3.1.9.7 for the multivariate ordinary least squares regression test (effect size f2 = 0.15, p err prob = 0.05, power = 0.8, and number of predictors = 9). This was exceeded with 307 complete survey responses, with extensive demographic representativeness (see results). To explore the relationship between NEP and level of agreeance with each problem statement/policy scenario, multivariate ordinary least squares regression models tested: (A) the effect of NEP on the level of agreeance with each problem statement/policy scenario; (B) the effect when controlling for relational values; (C) the effect when controlling for relational values, gender, age, years in sector, and occupation^2^. The model explaining the most variation was retained. No issues were identified with multicollinearity, heteroscedasticity or outliers; residuals were uncorrelated, and residuals were approximately normally distributed. The hypotheses are:

H_0_: There is no significant variation between NEP and how much participants agree with each problem statement/policy scenario.

H_a_: There is significant variation between NEP and how much participants agree with each problem statement/policy scenario.

### Interviews and qualitative data analysis

The interviews ranged from 29 to 49 min and were conducted online, recorded and transcribed. Interview protocols were designed to encourage reflection on the survey and the following themes: what is the problem in current livestock systems; future scenarios; research and policy priorities; influencing factors towards perspectives, and final remarks. Qualitative data was analysed using NVivo 12. Thematic codes were applied following a grounded theory methodology to produce *a posteriori* codes, i.e. developing theory from the data in an iterative/recursive process of data collection and analysis.

## Results

### Socio-demographic distribution

There were 307 complete survey responses balanced by age, gender, occupation and livestock sector (see Table 4). The majority (68.4%) of respondents were from the UK, followed by New Zealand (4.6%), the United States (3.6%) and Ireland (2.9%), but a total of 31 countries were represented. However, note that this study does not seek to be representative of each country, or find associations based on geographic location. The response rate to the qualitative survey questions was also high, with an average of 266 responses for each of the five questions.

**Table 4:** Socio-demographic distribution

	N	%
Age		
18–24	24	7.82
25–34	87	28.34
35–44	67	21.82
45–54	55	17.92
55–64	46	14.98
65–74	20	6.51
75–84	1	0.33
Prefer not to say	7	2.28
Gender		
Female	161	52.44%
Male	136	44.30%
Non-binary / third gender	1	0.33%
Other	2	0.65%
Prefer not to say	7	2.28%
Occupation		
Farmer	79	25.73
Researcher	77	25.08
Veterinarian	47	15.31
Postgraduate Student	33	10.75
Undergraduate Student	11	3.58
Producer	3	0.98
Processor	3	0.98
Other	54	17.59
Sector		
Beef	108	23.74
Sheep	106	23.30
Not sector specific	104	22.86
Dairy	72	15.82
Poultry	29	6.37
Other	36	7.91

In addition to the survey responses, a total of ten participants were interviewed from the 135 who agreed to a follow-up interview. These participants were systematically selected to represent a diversity of environmental worldviews, values, occupations, locations and other demographics. The NEP for those who agreed to be interviewed ranged from 15 to 60, with scores divided into high (46–60), medium (31–45) and low (15–30). Three participants were interviewed from high, four from medium, and three from low. The interviews included four researchers, three veterinarians, one farmer^3^, one postgraduate student and one ‘other’, representing most of the major roles engaged in the survey. 50% of interviewees were women and 50% men. Participants represented ages 25–34 (30%), 45–54 (20%), 55–64 (40%) and 65–74 (10%). Years involved in the livestock sector ranged from 3 to 50, with an approximately even distribution, and sectors represented included sheep, beef, dairy, poultry and non-sector specific. The interviews included participants from the UK, Belgium, Switzerland, New Zealand, United States and India. This systematic and purposive sampling approach successfully enabled the discovery of meaning from a diversity of perspectives (Given 2008, p.698), and it was felt that data saturation was achieved, i.e. further inquiry would add little to the story (Weiss 1994, p. 21).

### Overall perspectives towards the problem and solution

**Fig. 2:**
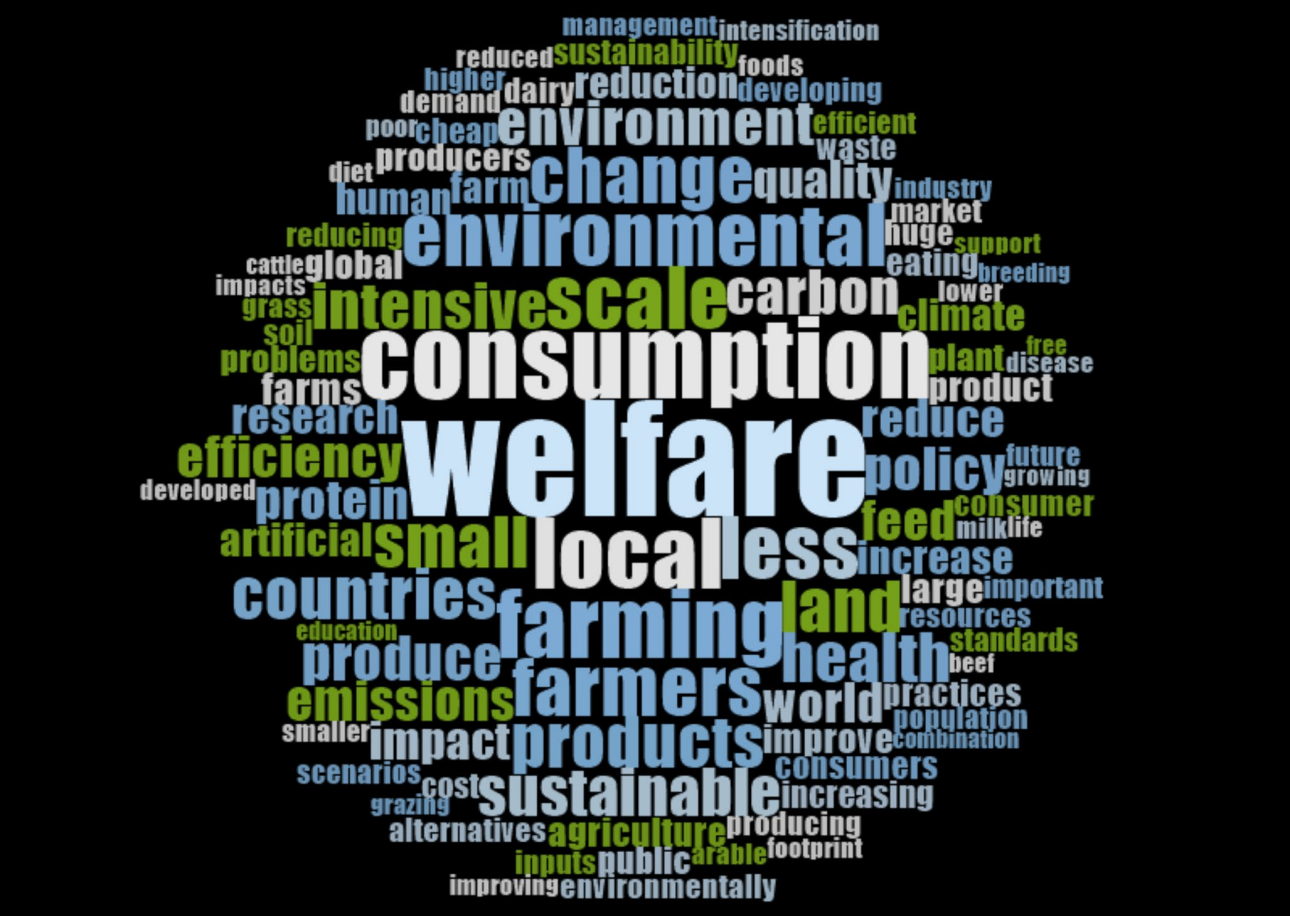
Most frequently mentioned words in the qualitative data. Words not meaningful to the context were removed. Minimum word length = 3

Survey participants mostly agreed that there is too much inequality (86.0%), and too much greed (72.5%) in current livestock systems – see Table 1 for statement definitions. However, participants were divided over whether they agreed or disagreed that there is not enough food– 44.1% agreed, 43.0% disagreed. Figure 2, showing the most frequent words in the qualitative survey responses and interview transcripts, revealed further perceived problems. These included (animal) welfare (308 mentions by 131 survey/interview participants), environment*^4^ (266 mentions, 144 participants), (over)consumption (210 mentions, 96 participants), intens* (151 mentions, 101 participants), (in)efficiency (143 mentions, 99 participants), health (120 mentions, 83 participants), policy (102 mentions, 32 participants), quality (86 mentions, 72 participants), pressures caused by ‘cheap’ meat (51 mentions, 50 participants) and waste (50 mentions, 38 participants).

Other problems highlighted in qualitative data included a disconnect between crop and livestock systems, a lack of circularity, lower standards for imported meat and an overuse of antibiotics. On the other hand, some participants felt that there is no problem in current systems, or that the problem was misunderstanding on the part of those outside the sector or ‘false information’ spread by activists and researchers. Finally, some participants emphasised the need to recognise complexity and variation within and between different livestock systems.

**Fig. 3:**
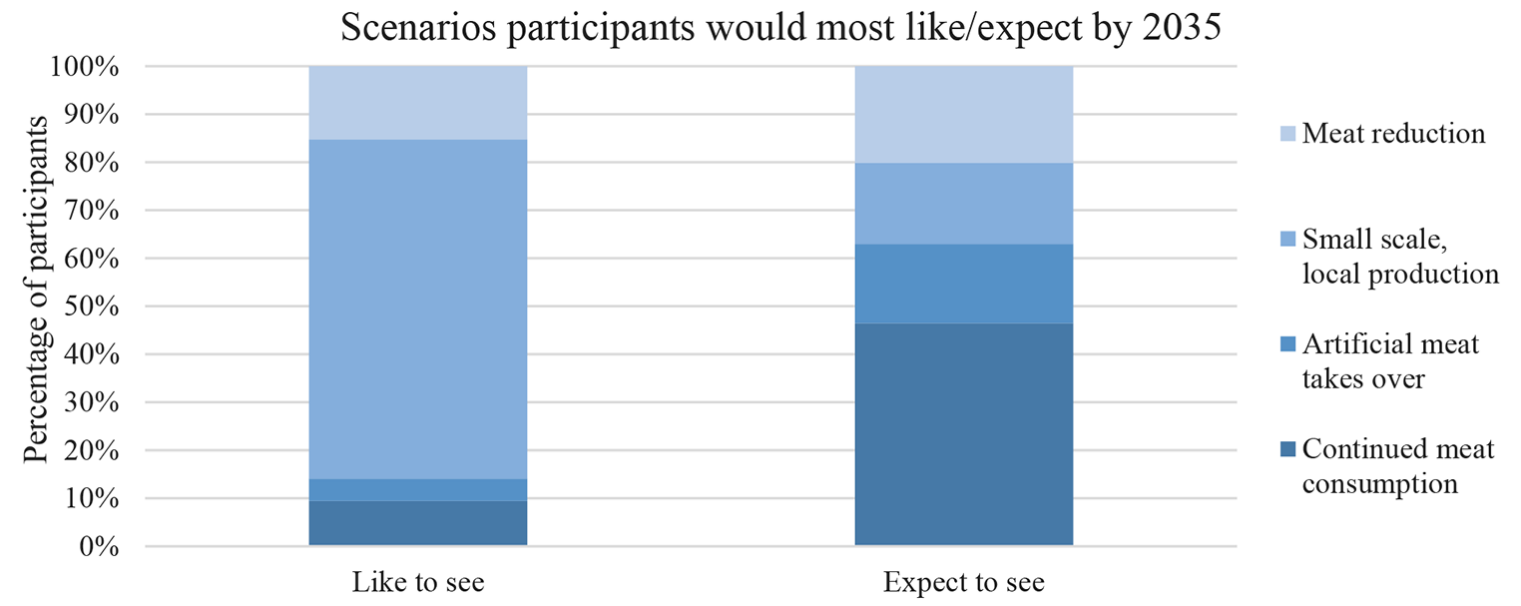
Livestock future scenarios participants would most like and expect to see by 2035

In relation to the livestock future scenarios, the majority of survey participants (70.6%) felt that the small scale, local production was the scenario they would most like to see by 2035, followed by meat reduction (15.3%), continued meat consumption (9.5%), and artificial meat (4.6%) – see Fig. 3. Interestingly, the story is very different for the scenario that participants expect to see by 2035. The majority instead expect to see continued meat consumption (46.5%), followed by meat reduction (20.2%), small scale, local production (16.8%) and artificial meat (16.5%).

Survey participants mostly agreed with the policy scenario statements associated with small scale, local production. The majority agreed that food production and distribution need to shift towards smaller scale, local systems of production, achieving self-sufficient food systems within countries should be a policy priority, and livestock solutions should focus on learning from traditional, indigenous and local knowledge − 82.6%, 78.5% and 69.1% respectively. Within meat reduction, the majority (52.4%) agreed that consumption in developed countries needs to drastically decline. However, the majority (59.3%) disagreed that governments should intervene, e.g. through livestock taxes or incentives for farmers to shift from animal rearing into other farming or non-farming activities. Almost all participants (93.4%) agreed that nutritional and environmental education should be a core element in the school curriculum. Within continued meat consumption, the majority (60.9%) disagreed that meat consumption patterns cannot and should not be moderated. However, the majority (59.3%) agreed that we need to increase the efficiency of meat production. The response to breeding and genetic modification for improving efficiency was more mixed − 44.2% agreed, 39.4% disagreed. Within artificial meat, the majority (60.6%) disagreed that there needs to be investment into developing artificial lab-grown meat. The response to investing in high protein foods derived from insects was more mixed − 38.8% agreed, 33.1% disagreed. More agreed (46.4%) with investing in high protein foods derived from plants and algae than disagreed (35.3%).

From the qualitative analysis, it was also clear that small scale, local production was popular, with ‘local’, ‘small’ and ‘scale’ being some of the most commonly mentioned words across the qualitative survey responses and interview transcripts (182, 127 and 158 mentions respectively). Participants discussed the importance of low food miles, self-sufficient food systems, food security, fair pricing, and trade deals that support local, high-quality produce with good welfare/environmental standards. However, responses were nuanced. For example, several participants discussed the benefits of local, including being able to control environmental/welfare standards, reducing animal welfare risks associated with long distance transport, and providing local development opportunities. However, an interviewee also pointed out that local does not necessarily equate with lower environmental impact, and that having lots of small-scale farms could add to the problem in terms of the environmental impacts of distribution and logistics.

Many participants also discussed the importance of reducing meat consumption in developed countries, with ‘less’, ‘reduce’ and ‘reduction’ featuring highly on the most commonly mentioned words (157, 87 and 68 respectively). The role of education, government/policy intervention, economic signals, meat tax and public procurement were discussed by participants – some more favourably than others. For example, some felt that education would not be sufficient without accompanying economic signals. Others discussed the unpopularity of a meat tax.

Participants also discussed benefits and drawbacks of intensifying and improving efficiency through technology, innovation, and breeding. ‘Intensive’, ‘efficient’, ‘intensification’ and ‘breeding’ received 114, 44, 37 and 33 mentions respectively. A few participants referred to the potential environmental benefits of intensification. However, an interviewee discussed how techno-fixes and an over-reliance on technology could be dangerous in reducing farmers’ stockmanship, i.e. their ability to understand animal behaviours and recognise problems, and could enable continued intensification with negative implications for animal welfare.

Lastly, artificial meat was widely discussed and divided opinions. ‘Artificial’ and ‘alternatives’ received 73 and 48 mentions respectively. Many participants discussed the potential benefits of meat alternatives and how we need to diversify our protein sources. A few felt that cell-cultured meat would be a novelty rather than a significant protein source. One interviewee considered the possible high environmental impact of cell-cultured meat. Another interviewee was concerned with the lack of welfare standards for insects in the growing insect-protein sector. Finally, another interviewee raised concern over unhealthy ultra-processed meat replacements, saying that that meat should be replaced with natural protein sources to protect our health.

An overwhelming finding from the qualitative data was a preference for a combination or balance of scenarios. These included behaviour-oriented approaches, those who favoured technological approaches, and those who favoured some aspects of technological and behaviour approaches.

### Environmental worldviews, values and demographics

**Fig. 4:**
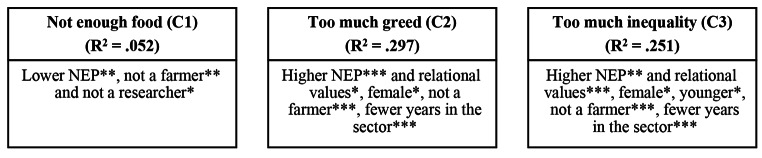
Effect of the New Ecological Paradigm (NEP) and control variables on perspectives towards the problem in the livestock sector. *** p < .01, ** p < .05, *p < .1. See Table 5 for details of the models

**Fig. 5:**
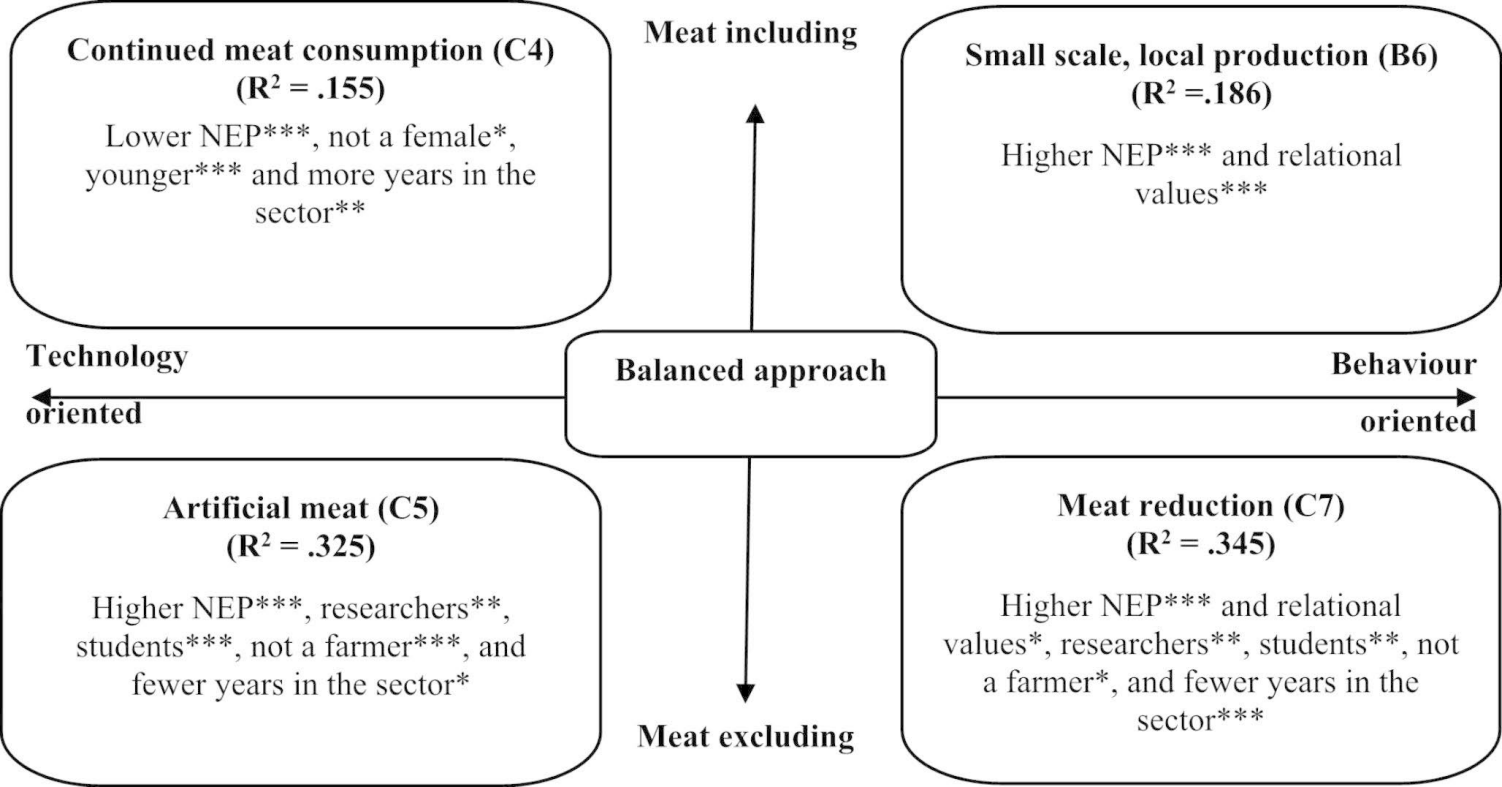
Effect of the New Ecological Paradigm (NEP) and control variables on perspectives towards livestock future policy scenarios. *** p < .01, ** p < .05, *p < .1. See Table 6 for details of the models

The results demonstrate the importance of environmental worldviews, values and demographic factors in influencing individual’s perspectives towards the future of the livestock sector. Results from the multivariate regression tests demonstrate significant variation between NEP and how much participants agree with each problem statement/policy scenario – see Figs. 4 and 5; Tables 5 and 6 - so the H_0_ can be rejected. The models explained the most variation (the highest R^2^) when controlling for relational values, gender, age, years in sector and occupation (models C1-5 and C7), except for ‘small scale, local production’. In that case, controlling for just relational values offered the best explanation (model B6). All but one of the models were able to explain between 16 and 35% of variation. R^2^ values in this range are very common in trying to predict human behaviour given that social systems are complex. We can therefore draw important conclusions from the statistically significant predictors. One model - ‘Not enough food’ (C1) – had a lower R^2^, explaining 5.2% of variation, so though the statistically significant predictors are still valid, we should be cautious in overstating the explanatory power of the model.

For the problem statements, those with higher NEP scores were significantly more likely to agree with too much greed and inequality (p < .01, 0.05 respectively). Whereas those with lower NEP scores were significantly more likely to agree with not enough food (p < .05). Higher relational values also had a significant effect on too much greed and equality (p < .1, 0.01 respectively). Many of the demographic control factors also had a significant effect. For example, females were significantly more likely to agree with too much greed and equality (p < .1). Younger participants were significantly more likely to agree with too much inequality (p < .1). Farmers were significantly less likely to agree with all three problem statements (p < .01 for too much greed/inequality and p < .05 for not enough food). Researchers were also less likely to agree that there is not enough food (p < .1). Finally, those with fewer years in the sector were significantly more likely to agree with too much greed and equality (p < .01).

**Table 5:** Multivariate analysis of the New Ecological Paradigm (NEP) and control variables on perspectives towards the problem in the livestock sector

Independent variables ^a^	Dependent variables / models								
	Not Enough Food			Too Much Greed			Too Much Inequality		
	A1 ^b^	B1 ^b^	C1 ^b^	A2 ^b^	B2 ^b^	C2 ^b^	A3 ^b^	B3 ^b^	C3 ^b^
NEP	− 0.019** **(0.036)**	− 0.021** (0.039)	− 0.026** **(0.020)**	0.068*** (0.000)	0.058*** (0.000)	0.051*** **(0.000)**	0.033*** (0.000)	0.020*** (0.002)	0.016** **(0.019)**
RV		0.009 (0.655)	0.001 (0.953)		0.047*** (0.005)	0.033* **(0.054)**		0.057*** (0.000)	0.040*** **(0.002)**
Gender (Female = 1)			0.056 (0.752)			0.251* **(0.073)**			0.185* **(0.089)**
Age			− 0.093 (0.226)			0.050 (0.410)			0.088* **(0.064)**
Years in sector			0.009 (0.195)			− 0.015*** **(0.006)**			− 0.012*** **(0.004)**
Occupation (Famer = 1)			− 0.657** **(0.010)**			− 0.541*** **(0.007)**			− 0.481*** **(0.002)**
Occupation (Researcher = 1)			− 0.412* **(0.097)**			− 0.260 (0.184)			0.209 (0.169)
Occupation (Student = 1)			− 0.441 (0.133)			− 0.144 (0.599)			0.067 (0.708)
Occupation (Veterinarian = 1)			− 0.314 (0.259)			− 0.322 (0.141)			0.112 (0.510)
Constant	2.825*** **(0.000)**	2.711*** **(0.000)**	**3.511** **(0.000)**	− 0.160 (0.624)	− 0.764** (0.049)	0.168 (0.711)	1.944*** **(0.000)**	1.211*** **(0.000)**	1.762***a **(0.000)**
R^2^	0.014	0.015	0.052	0.208	0.228	0.297	0.086	0.137	0.251
Observations	312	312	291 ^c^	312	312	291 ^c^	312	312	291 ^c^

^*a*^*Testing on multicollinearity shows independence between predictors*.

^*b*^ *Models show: (A) the effect of the New Ecological Paradigm score (NEP) on level of agreeance with each problem statement; (B) the effect when controlling for the relational values score (RV); and (C) the effect when controlling for RV, gender, age, years in sector and occupation.*

^*c*^*Number of observations are lower for model C due to fewer survey responses to demographic questions*.

*** p < .01, ** p < .05, *p < .1. Unstandardised coefficients are reported with standard errors reported in parentheses.

For the policy scenarios, those with higher NEP scores were significantly more likely to agree with small scale, local production, artificial meat and meat reduction (p < .01). Whereas those with lower NEP scores were significantly more likely to agree with continued meat consumption (p < .01). Higher relational values also had a significant effect on small scale, local production and meat reduction (p < .01, p < .1 respectively). Many of the demographic control factors also had a significant effect. For example, those not identifying as female, and younger participants were significantly more likely to agree with continued meat consumption (p < .1, 0.01 respectively). Farmers were significantly less likely to agree with the meat excluding scenarios (p < .01 for artificial meat, p < .1 for meat reduction). Whereas researchers and students were more likely to agree with meat excluding scenarios (p < .01 for students/artificial meat, p < .05 for researchers/artificial meat and researchers/students/meat reduction). Finally, those with fewer years in the sector were significantly more likely to agree with artificial meat and meat reduction (p < .1, 0.01 respectively) and those with more years in the sector were significantly more likely to agree with continued meat consumption (p < .05).

**Table 6:** Multivariate analysis of the New Ecological Paradigm (NEP) and perspectives towards livestock future policy scenarios

Independent variables ^a^	Dependent variables / models												
	Continued Meat Consumption			Artificial Meat			Small Scale Local Production			Meat Reduction			
	A4 ^b^	B4 ^b^	C4 ^b^	A5 ^b^	B5 ^b^	C5 ^b^	A6 ^b^	B6 ^b^	C6 ^b^	A7 ^b^	B7 ^b^	C7 ^b^	
NEP	− 0.102*** **(0.000)**	− 0.102*** (0.000)	− 0.097*** **(0.000)**	0.123*** (0.000)	0.105*** (0.000)	0.119*** **(0.000)**	0.079*** (0.000)	0.042*** (0.004)	0.022 (0.154)	0.133*** (0.000)	0.106*** (0.000)	0.108*** **(0.000)**	
RV		− 0.001 (0.983)	− 0.003 (0.944)		0.081* (0.082)	0.010 (0.812)		0.164*** (0.000)	0.173*** **(0.000)**		0.121*** **(0.000)**	0.063* **(0.065)**	
Gender (Female = 1)			− 0.597* **(0.089)**			− 0.164 (0.651)			0.213 (0.396)			0.145 (0.603)	
Age			− 0.493*** **(0.001)**			− 0.096 (0.542)			0.093 (0.393)			0.140 (0.249)	
Years in sector			0.028** **(0.043)**			− 0.026* **(0.067)**			− 0.009 (0.354)			− 0.042*** **(0.000)**	
Occupation (Famer = 1)			− 0.729 (0.148)			-1.76*** **(0.001)**			0.547 (0.130)			− 0.740* **(0.066)**	
Occupation (Researcher = 1)			− 0.049 (0.623)			1.239** **(0.015)**			− 0.041 (0.907)			0.902** **(0.022)**	
Occupation (Student = 1)			− 0.639 (0.272)			2.022*** **(0.001)**			0.079 (0.850)			0.977** **(0.036)**	
Occupation (Veterinarian = 1)			− 0.277 (0.614)			0.625 (0.271)			− 0.022 (0.955)			0.646 (0.142)	
Constant	10.266 (0.000)	10.277 (0.000)	11.264 (0.000)	0.203 (0.824)	− 0.847 (0.438)	0.784 (0.507)	6.020 (0.000)	3.898 (0.000)	4.264 (0.000)	1.754 (0.010)	0.187 (0.816)	1.579 (0.084)	
R^2^	0.095	0.095	0.155	0.099	0.107	0.325	0.099	0.186	0.181	0.187	0.218	0.345	
Observations	312	312	291 ^c^	312	312	291 ^c^	312	312	291 ^c^	312	312	291 ^c^	

^*a*^*Testing on multicollinearity shows independence between predictors*.

^*b*^ *Models show: (A) the effect of the New Ecological Paradigm score (NEP) on level of agreeance with each of the policy scenarios; (B) the effect when controlling for the relational values score (RV); and (C) the effect when controlling for RV, gender, age, years in sector and occupation.*

^*c*^*Number of observations are lower for model C due to fewer survey responses to demographic questions*.

*** p < .01, ** p < .05, *p < .1. Unstandardised coefficients are reported with standard errors reported in parentheses.

## Discussion

### Environmental worldviews, values, demographics and perspectives

This study has demonstrated the importance of environmental worldviews, values and underlying demographic factors in influencing individual’s perspectives towards the future of the livestock sector, including perspectives towards the problem and policy scenarios. This understanding is crucial because it can inform policy design that can effectively influence behaviours within the sector and break the cycle of inertia. It will also enable more appropriate framing of messages and approaches to the different perspectives to promote sustainable livestock futures. By acknowledging the complexity of individual perspectives and the various factors that influence them, policymakers can develop targeted strategies that can lead to meaningful change within the livestock sector.

Given that high NEP scores have been shown to be associated with pro-environmental values and ecocentrism (Hunter and Rinner 2004; Kortenkamp and Moore 2001; Gangaas et al. 2015), and low NEP scores have been shown to be associated with technocentrism (Gerhard 2004; Gangaas et al. 2015) we can make further inferences about the results. Those with higher pro-environmental, ecocentric and relational worldviews and values are more likely to consider the problem being too much inequality and greed in current systems, favouring more behaviour-oriented (and artificial meat^5^) solutions. In contrast, those with lower pro-environmental and higher technocentric worldviews and values are more likely to think that there is not enough food in current systems, and that we should continue to increase global meat consumption and invest in technological solutions to improve efficiency of meat production.

Overall, the role of environmental worldviews and values in influencing livestock future perspectives is not surprising given that ecocentric perspectives emphasise equitable redistribution of resources and environmental protection, technocentric perspectives emphasise systemic continuity and scientific/technological solutions to environmental challenges (Dryzek 2013), and relational values represent strong connections to other people, nature and animals, and core values relating to care, justice, reciprocity and virtue (Chan et al. 2016). The link between low pro-environmental worldviews and values and the continued meat consumption pathway that is resistant to change and depends on technocentric solutions to enhance efficiency could be interpreted as a reflection of the productionist worldview, which values productivity and efficiency above environmental and social considerations (Bawden, 2006; Page and Witt 2022). The study’s finding that less than 10% of participants agreed with this productionist framing is a promising indication of a shift in perspectives towards alternative solutions to sustainability challenges in the livestock sector, and a growing recognition of the negative impacts of the dominant worldview. However, the study also revealed a hint of pessimism as nearly half of the participants expected the continued meat consumption pathway to persist. This echoes the sentiment expressed by policymakers and commentators in the NFS (2021) report, who were sceptical or even defeatist about the possibility of radical change in the food system. Further investigation is necessary to comprehend the underlying factors that contribute to pessimistic attitudes towards the feasibility of alternative pathways in the sector.

The findings also highlighted the significant influence of demographic factors on perspectives, including age, gender, occupation, and years of experience in the sector. That younger people were more likely to agree with the continued meat consumption policy scenario was surprising given that previous studies have shown that younger people tend to be more environmentally conscious (Diamantopoulos et al. 2003), and are more likely to eat less meat (Ruby 2012; Charlebois et al. 2020; Gossard and York 2003). However, the fact that individuals who do not identify as female in this study were more likely to agree with the continued meat consumption policy scenario aligns with previous studies, e.g. showing men to be less positive about reducing meat consumption than women (Ruby and Heine 2011; Rosenfield, 2020; Schösler et al. 2015; Judge and Wilson 2019; Prättälä et al. 2007; Rothgerber 2013; Kubberød et al. 2002). That researchers and students were more likely to support meat excluding scenarios also corresponds to previous studies showing that those with higher education tend to eat less meat (Mata et al. 2022; Gossard and York 2003; Rimal 2002). Finally, that livestock farmers were more likely to disagree with meat excluding scenarios is unsurprising and corresponds to previous studies where individuals whose livelihoods depend on agriculture tend to prioritise and place higher value on agricultural resources (Wardropper, 2020) and those in labourer occupations, including farmers, tend to eat more meat (Gossard and York 2003).

### Policy insights

When formulating specific policy measures and future pathways for sustainable livestock systems, it is imperative to consider the plurality of worldviews and values held by various stakeholders. For example, the popularity of small-scale, local production systems uncovered in this study could reflect a growing interest in community-based approaches to food production and consumption, prioritising environmental protection, animal welfare, and diversification. However, small scale, extensive systems might not always equate with better environmental outcomes. The ‘land sparing’ versus ‘land sharing’ debate reflects some of the competing worldviews and values in this space. Land sparing suggests that intensive production systems can be more efficient and spare more land for carbon-offset/biodiversity through conservation, rewilding, tree planting and nature reserves (Balmford, 2021; Fischer et al. 2014). Whereas, the land-sharing advocates extensive livestock systems that incorporate techniques that increase biodiversity and carbon uptake (Balmford, 2021; Fischer et al. 2014). The UK National Food Strategy report recommends a mosaic of land uses, including semi-natural land, high-yield farmland, and low-yield farmland, to optimise environmental outcomes, such as reducing GHG emissions and promoting biodiversity (NFS, 2021). Incorporating a better understanding of the diverse worldviews, values, and cultural factors will be crucial to improving the efficacy of such land use plans. Such recognition could help to identify potential trade-offs and synergies between different policy options, facilitating more informed, inclusive and effective decision-making for sustainable agro-food systems.

The survey offered further insights into stakeholders’ values and priorities, for example, the promotion of self-sufficiency in food systems was a very popular policy measure amongst all stakeholder categories, indicating strong values relating to autonomy and independence. Self-sufficiency in food systems helps to build strong domestic farming sectors, and protects countries from international supply disruptions, for example, during war/political tension, sudden rises in food prices, or production shortfalls internationally (Clapp 2017; FAO, 1996). However, it is important to note that self-sufficiency does not guarantee food security so we must also consider and plan for potential local food crises (Zurek et al. 2022). Additionally, while some view policy measures such as trade tariffs, export bans, and subsidies as necessary to support rural communities and economic development goals, others argue that they can distort markets (Clapp 2017). In this study, many discussed the importance of trade deals that support local, high-quality produce with good welfare and environmental standards.

Furthermore, it was promising to see the recognition of alternative indigenous worldview and value systems, with most survey respondents recognising the importance of traditional, indigenous and local knowledge. There is a wide range of traditional, indigenous and local practices in livestock systems that can help improve environmental and animal health outcomes, for example ethno veterinary medicine, methods to increase carbon sinks, agroforestry/intercropping with livestock, soil/water conservation, and breed selection (Abraha 2016; Ramos-Castillo and Galloway-McLean 2012; Van den Ban et al. 1995; Rajasekaran 1993). These practices importantly offer place-based solutions, which could be adapted and applied in other contexts to replace or supplement modern technological solutions and to generate ideas for further research (Van den Ban et al. 1995). However, there is a risk of losing traditional knowledge if it is not valued, documented and passed on, particularly if practices are displaced by perspectives that deem them to be ‘unscientific’ or ‘primitive’ (Van den Ban et al. 1995, p.122; Raygorodetsky 2011).

The meat reduction scenario generated mixed reactions amongst stakeholders in this study, demonstrating differences in priorities, worldviews and values. Some policies were well-received, including nutritional and environmental food education. The NFS (2020) also found education to be the most popular policy measure, empowering young people and the wider population with knowledge on food, nutrition, production, provenance, and cooking skills. These findings align with the values of informed decision-making and empowerment. However, government intervention through taxes or incentives for farmers to shift from animal rearing into other farming or non-farming activities was an unpopular policy measure in this study. These findings are consistent with the NFS (2021, p.122) where a tax on fresh meat was unpopular. Though the NFS found fewer people opposed a tax on processed meat, and other policy measures like targets for supermarkets/fast food chains to reduce meat sales were also popular, indicating that these interventions might be preferred if they are targeted elsewhere in the supply chain. The opposition to a meat tax may be driven by various factors, including values related to individual choice and freedom or cultural values attached to meat consumption. Moreover, some stakeholders may understandably prioritise their own job security in the livestock sector, which can lead to scepticism towards meat reduction pathways. Conversely, other stakeholders may be more aligned with meat reduction pathways due to their employment in industries that do not depend on meat production, or values regarding environmental sustainability, public health, and animal welfare.

Perspectives towards technology-oriented solutions were also mixed. One of the least popular policy measures was investing in lab-grown meat. Investing in plant- and algae-based proteins were more popular, and perspectives towards insect-derived proteins were mixed. Some previous studies have shown high consumer acceptance of lab-grown and plant-based alternatives (Bryant et al. 2019; Flycatcher, 2013), whereas others have shown low acceptance compared to conventional meat (Slade 2018; Hocquette et al. 2015; Verbeke et al. 2015; Wilks and Phillips 2017). Nomenclature, i.e. the names given to alternative meats, has also been shown to significantly influence attitudes e.g. ‘clean meat’ results in more positive attitudes than ‘lab-grown meat’ and ‘cultured meat’ (Bryant and Barnett 2019; ACE, 2017; GFI, 2017). Openness to entomophagy (eating insects) has also been shown to be mixed and highly context dependent (Menozzi et al. 2017; Lensvelt and Steenbekkers 2014). Furthermore, the acceptance and uptake of technology, including lab-grown meat and plant-based alternatives, may be influenced by various competing worldviews and values. For example, Schwartz (2012) identifies a tension between the competing values of ‘openness to change’ and ‘conservation’ (i.e. resistance to change). In this context, openness to change could align with values of innovation, progress, and embracing new technologies that promise to address pressing sustainability challenges. On the other hand, conservation values may prioritise the protection of traditional food systems and cultural practices. The use of technology in food production, such as lab-grown meat, also raises important ethical and moral considerations that must be taken into account. For instance, some individuals may view lab-grown meat as a more humane alternative to traditional animal agriculture, while others may be opposed to the manipulation of natural systems and the potential unknown consequences of such technologies (Bhat et al. 2019).

Perspectives towards breeding and genetic modification in this study were also mixed, with some stakeholders seeing the potential benefits for the environment, public and animal health, and others perceiving it as being potentially dangerous for animal welfare. Again, it is important to recognise that these perspectives are shaped by worldviews and values that influence individual perceptions of risks and benefits. A review by de Graeff et al. (2019) found that although there have been many proven benefits of genomic technology, there is a need to increase disciplinary diversity and representation of animal interests in research, to conduct systemic comparisons of potential impacts of the technologies, and to increase public engagement. Such measures could help to address the concerns and values held by different stakeholders, fostering more inclusive and sustainable practices in the livestock sector.

In the UK context, various balanced approaches have been recommended to address sustainability in the livestock sector, reflecting different values and priorities. For example, the NFS (2021), Climate Change Committee (2020) and Food, Farming and Countryside Commission (2021) proposed a mix of technological and behavioural interventions to address both the consumption and production of livestock, meanwhile the National Farmers Union (2019) propose technological interventions to increase productivity and carbon sequestration but with no behavioural change in diets. Careful consideration should be given to the potential impact of proposals, taking into account factors such as cultural and geographical contexts, environmental and human health implications, and animal welfare concerns. To ensure effective policy implementation, it is also crucial to address the diverse needs and priorities of all stakeholders involved, including not only the livestock representatives studied in this research, but also the general public, consumers, non-governmental organisations, and other relevant stakeholders. To promote effective decision-making and policy implementation, it is essential to address conflicting perspectives regarding the desirability of future scenarios in the livestock sector.

### Implications for the future of the livestock sector

The study findings suggest that the future of the livestock sector will be shaped by divergent perspectives, values, and priorities. These findings are consistent with previous research that has identified the agro-food system as a site of conflict between different paradigms, worldviews, and values, including productionism and more ecologically-integrated approaches (Bawden, 2006; Lang and Heasman 2015; Godfray 2015; Zaremba et al. 2021; Page and Witt 2022). This study and previous literature have demonstrated that transforming livestock futures will require recognising and reconciling the plurality of worldviews and values, promoting their exchange and convergence, and developing holistic understandings of human-nature interdependence (Bawden, 2006; Cayre et al. 2018; Plumecocq et al. 2018; Laininen 2019; El Bilali 2019; Dumont et al. 2021; Page and Witt 2022). Failure to do so may lead to further polarisation and entrenched positions, making it more difficult to develop and implement effective solutions that are acceptable to all stakeholders involved.

Adopting a pluralistic and pragmatic approach that considers diverse worldviews, values, trade-offs, and biological, social, and cultural values, as suggested by Robinson (2011), could benefit the development of more equitable decision-making and sustainable agro-food and livestock systems. Transformation will also require a profound systemic values transformation and a shift towards more systemic and holistic approaches to agricultural development. While transforming the livestock sector is challenging, history has shown that worldviews and values can change dramatically, as seen in changing attitudes towards smoking, gay rights, and whaling (Bergstrand 2014). Social movements can use ‘cognitive shocks’, where new information challenges previously held beliefs, to achieve transformative change in the livestock sector, mobilising supporters around new ideas and perspectives (Bergstrand 2014). This approach links new knowledge to specific actions and encourages people to participate, converting sentiments into acts of activism. However, resistance and scepticism from individuals whose beliefs are challenged must be addressed for the approach to be effective. This study’s findings therefore have important implications for effective communication and engagement with stakeholders. By understanding their worldviews and values, messages can be framed to resonate with their beliefs and attitudes, increasing motivation to achieve transformative change.

### Methodological reflections

The mixed methods approach was particularly effective in uncovering the processes involved in the development of environmental worldviews, values and perspectives and towards the future of the livestock sector, and in drawing out nuance. The NEP proved to be effective in quantifying participant’s environmental worldviews and values. Despite being the most widely used measure of environmental attitudes, the NEP has faced criticism, e.g. for being individualist, unidimensional, lacking standardisation, and for not covering all aspects of environmentalism (Bernstein 2020); though Bernstein (2020) also notes that data triangulation can improve measures of environmentalism. The inclusion of relational value statements, qualitative survey responses and interviews have certainly helped to produce a more pluralistic representation of environmental worldviews and values.

To date, most empirical work on relational values has used qualitative research methods due to the perceived risk of generalisation and appropriation by external actors (Schulz and Martin-Ortega 2018). This study took a mixed methods approach to relational values, incorporating a survey and interviews. Mixing methods in this way can improve the empirical evidence base for testing hypotheses, enable the ‘discovery’ of unifying elements across different cultures and can improve political legitimacy through improved representation of views (Schulz and Martin-Ortega 2018). The survey also provided further evidence that relational values are distinct influencing factors compared to the NEP scale, as in Klain et al. (2017), further justifying this approach.

While this study’s framing of worldviews and values was necessary to draw concrete conclusions about the relationship between these factors and perspectives in the livestock sector, it is important to consider alternative theoretical frameworks in future research. Adopting pluralistic and relational theoretical frameworks, such as pragmatism, ecofeminism, or more-than-human geographies, could offer a more holistic and inclusive understanding of worldviews and values, and better capture the complex dynamics involved in shaping the future of the livestock sector. Incorporating such approaches could lead to a more nuanced and comprehensive understanding of the relationship between worldviews, values, and perspectives in the context of the livestock sector.

## Conclusions

Despite widespread consensus on the need for change, transformation in the livestock debate has been characterised by polar opinions and inertia. This study has demonstrated the importance of environmental worldviews, values and demographic factors in influencing perspectives towards the future of the livestock sector. For example, those with higher pro-environmental, ecocentric and relational worldviews and values favour more behaviour-oriented solutions. Those with lower pro-environmental and higher techno-centric worldviews and values favour technological solutions to improve the efficiency of production and to enable continued patterns of meat consumption. Understanding this relationship will be important in the formulation of livestock policy and can enable more appropriate framing of messages and approaches to the different perspectives to promote sustainable livestock futures.

The aim of this study was not to determine the best approach to transformation, but rather to foster an understanding of the processes that lead to diverse perspectives and to enable development of more holistic solutions that acknowledge all voices in an increasingly polarised debate. Communication between perspectives has often been unsuccessful because of contrasting worldviews, values, ways of life and solution strategies. Although the different perspectives are often framed as rivals, we need a plurality of worldviews and values in balance to achieve sustainability, given that the different approaches are interdependent, complementary and highlight the others’ limitations (Chuang et al. 2020; Thompson et al. 1990). The sustainability challenges associated with the livestock sector can usefully be deemed ‘wicked problems’, therefore solutions following a singular worldview framing of the problem/solution are no longer satisfactory (Grint 2010; Chuang et al. 2020). Adopting more pluralistic, relational methodologies will therefore be paramount in developing solutions for sustainable livestock futures.

## Footnotes

1 CO_2_e, or carbon dioxide equivalent, is the standard unit for carbon footprint measurement. It reflects CO_2_ and other greenhouse gases in terms of the volume of CO_2_ that would create the same warming effect.

2 For occupation, producers and processors were excluded from the regression model due to a small sample size. ‘Other’ was excluded due to substantial variation in occupations represented e.g. from crofters, to animal welfare consultants, to laboratory technicians and many more, so no meaningful conclusion could be drawn from these results. Finally, postgraduate and undergraduate students were combined into a single ‘student’ variable to increase the sample size.

3 A total of six farmers were contacted but only one responded. Future work will seek to represent more farmer perspectives through interviews/focus groups. However, 79 farmers (25.7% of the total) completed the survey and gave written qualitative feedback.

4 * represents a wildcard used to represent any number of characters. So for example, environment* could include environment, environments, environmental, etc.

5 NB: Relational values were not significantly higher for artificial meat.

## References

[CR1] Abraha, F. 2016. *Indigenous livestock husbandry and ethno veterinary practices in Endamohoni District of Tigray Region, Ethiopia*. Doctoral dissertation, Hawassa University.

[CR2] ACE (Animal Charity Evaluators). 2017. *“Clean” meat or “cultured” meat: A randomized trial evaluating the impact on self-reported purchasing preferences*. [Online]. [Accessed 10 January 2023]. Available from: https://animalcharityevaluators.org/blog/clean-meat-or-cultured-meat-a-randomized-trial-evaluating-the-impact-on-self-reported-purchasing-preferences/.

[CR3] Adesogan, A. T., A. H. Havelaar, S. L. McKune, M. Eilittä, and G. E. Dahl. 2020. Animal source foods: sustainability problem or malnutrition and sustainability solution? Perspective matters. *Global Food Security, 25*, p.100325.

[CR4] Alexander P, Brown C, Arneth A, Finnigan J, Moran D, Rounsevell MD (2017). Losses, inefficiencies and waste in the global food system. Agricultural systems.

[CR5] Balmford, A. 2021. Concentrating vs. spreading our footprint: how to meet humanity’s needs at least cost to nature. *Journal of Zoology, 315*(2), pp.79–109.Bawden, R., 2006. A systemic evaluation of an agricultural development: A focus on the worldview challenge. In *Systems concepts in evaluation* (pp.35–46). Point Reys, CA: Edge Press of Inverness.

[CR6] Bergstrand K (2014). Cognitive shocks: Scientific Discovery and mobilization. Science as Culture.

[CR7] Bernstein J (2020). Dis) agreement over what? The challenge of quantifying environmental worldviews. Journal of Environmental Studies and Sciences.

[CR8] Bhat ZF, Morton JD, Mason SL, Bekhit AEDA, Bhat HF (2019). Technological, regulatory, and ethical aspects of in vitro meat: a future slaughter-free harvest. Comprehensive Reviews in Food Science and Food Safety.

[CR10] Bryant CJ, Barnett JC (2019). What’s in a name? Consumer perceptions of in vitro meat under different names. Appetite.

[CR9] Bryant, C., K. Szejda, N. Parekh, V. Desphande, and B. Tse. 2019. A survey of consumer perceptions of plant-based and clean meat in the USA, India, and China. *Frontiers in Sustainable Food Systems, 3*, p.11.

[CR11] Cassidy ES, West PC, Gerber JS, Foley JA (2013). Redefining agricultural yields: from tonnes to people nourished per hectare. Environmental Research Letters.

[CR12] Cayre, P., A. Michaud, J. P. Theau, and C. Rigolot. 2018. The coexistence of multiple worldviews in livestock farming drives agroecological transition. A case study in French Protected Designation of Origin (PDO) cheese mountain areas. *Sustainability, 10*(4), p.1097.

[CR13] CCAC (Climate and Clean Air Coalition). 2020. *The Climate and Clean Air Coalition 2030 Strategy*. [Online]. [Accessed 6 January 2023]. Available from: https://www.ccacoalition.org/en/resources/climate-and-clean-air-coalition-2030-strategy.

[CR14] CCC (Climate Change Committee). 2020. *Sixth carbon budget*. [Online]. [Accessed 10 January 2023]. Available from: https://www.theccc.org.uk/publication/sixth-carbon-budget/.

[CR15] Chan, K. M., P. Balvanera, K. Benessaiah, M. Chapman, S. Díaz, E. Gómez-Baggethun, R. Gould, N. Hannahs, K. Jax, S. Klain, and G. W. Luck. 2016. Opinion: Why protect nature? Rethinking values and the environment. *Proceedings of the national academy of sciences, 113*(6), pp.1462–1465. 10.1073/pnas.1525002113 PMC4760809 26862158

[CR16] Charlebois S, Somogyi S, Music J, Caron I (2020). Planet, Ethics, Health and the New World Order in Proteins. Journal of Agricultural Studies.

[CR17] Christen, C. 2021. *Investigation: How the Meat Industry is Climate-Washing its Polluting Business Model* [Online]. [Accessed 10 January 2023]. Available from: https://www.desmog.com/2021/07/18/investigation-meat-industry-greenwash-climatewash/.

[CR18] Chuang, F., E. Manley, and A. Petersen. 2020. The role of worldviews in the governance of sustainable mobility. *Proceedings of the National Academy of Sciences, 117*(8), pp.4034–4042. 10.1073/pnas.1916936117 PMC7049160 32034101

[CR19] Clapp J (2017). Food self-sufficiency: making sense of it, and when it makes sense. Food policy.

[CR20] de Graeff, N., K. R. Jongsma, J. Johnston, S. Hartley, and A. L. Bredenoord. 2019. The ethics of genome editing in non-human animals: a systematic review of reasons reported in the academic literature. *Philosophical Transactions of the Royal Society B, 374*(1772), p.20180106. 10.1098/rstb.2018.0106 PMC6452271 30905297

[CR21] Diamantopoulos A, Schlegelmilch BB, Sinkovics RR, Bohlen GM (2003). Can socio-demographics still play a role in profiling green consumers? A review of the evidence and an empirical investigation. Journal of Business research.

[CR22] Dryzek JS (2013). The politics of the earth: environmental discourses.

[CR23] Dumont, A. M., A. C. Wartenberg, and P. V. Baret. 2021. Bridging the gap between the agroecological ideal and its implementation into practice. A review. *Agronomy for sustainable development, 41*(3), p.32.

[CR24] Dunlap REVL, Liere KV, Mertig A, Jones RE (2000). Measuring endorsement of the new ecological paradigm: a revised NEP scale. Journal of social issues.

[CR25] Dunne, D. 2021. *Global meat industry ‘using tobacco company tactics’ to downplay role in driving climate crisis, investigation claims* The Independent. [Online]. [Accessed 10 January 2023]. Available from: https://www.independent.co.uk/climate-change/news/meat-dairy-industry-greenwashing-climate-b1884769.html.

[CR26] El Bilali, H. 2019. The multi-level perspective in research on sustainability transitions in agriculture and food systems: A systematic review. *Agriculture, 9*(4), p.74.

[CR27] European Commission. 2013. *The Impact of EU Consumption on Deforestation: Comprehensive analysis of the impact of EU consumption on deforestation* Technical Report 2013–063.

[CR28] FAO (Food and Agriculture Organization of the United Nations). 1996. *Food and International Trade Technical Background Document* [Online]. [Accessed 10 January 2023]. Available from: http://www.fao.org/docrep/003/w2612e/w2612e12.htm.

[CR29] FAO (Food and Agriculture Organization of the United Nations) (2013). Tackling climate change through livestock – a global assessment of emissions and mitigation opportunities.

[CR30] FFCC (Food, Farming and Countryside Commission). 2021. *Modelling an agroecological UK in 2050* IDDRI for FFCC. [Online]. [Accessed 10 January 2023]. Available from: https://www.iddri.org/sites/default/files/PDF/Publications/Catalogue%20Iddri/Etude/202111-ST1021-TYFA%20UK_0.pdf.

[CR31] Fischer J, Abson DJ, Butsic V, Chappell MJ, Ekroos J, Hanspach J, Kuemmerle T, Smith HG, von Wehrden H (2014). Land sparing versus land sharing: moving forward. Conservation Letters.

[CR32] Flycatcher., 2013. *Kweekvlees [Cultured meat]* [Online]. [Accessed 10 January 2023]. Available from: http://www.flycatcherpanel.nl/news/item/nwsA1697/media/images/Resultaten_onderzoek_kweekvlees.pdf.

[CR33] Gallet CA (2010). Meat meets meta: a quantitative review of the price elasticity of meat. American Journal of Agricultural Economics.

[CR34] Gangaas KE, Kaltenborn BP, Andreassen HP (2015). Environmental attitudes associated with large-scale cultural differences, not local environmental conflicts. Environmental Conservation.

[CR35] Garnett T (2015). Gut feelings and possible tomorrows: (where) does animal farming fit.

[CR36] Gerhard LC (2004). Climate change: conflict of observational science, theory, and politics. AAPG Bulletin.

[CR37] GFI (Global Food Institute). 2021. *Record $3.1 billion invested in alt proteins in 2020 signals growing market momentum for sustainable proteins*. [Online]. [Accessed 10 January 2023]. Available from: https://gfi.org/blog/2020-state-of-the-industry-highlights/.

[CR38] Given LM (2008). The SAGE encyclopedia of qualitative research methods.

[CR39] Godfray HCJ (2015). The debate over sustainable intensification. Food Security.

[CR40] Godfray, H. C. J., P. Aveyard, T. Garnett, J. W. Hall, T. J. Key, J. Lorimer, R. T. Pierrehumbert, P. Scarborough, M. Springmann, and S. A. Jebb. 2018. Meat consumption, health, and the environment. *Science, 361*(6399). 10.1126/science.aam5324 30026199

[CR41] Gossard, M. H., and R. York. 2003. Social structural influences on meat consumption. Human Ecology Review, pp.1–9.

[CR42] Grint K (2010). Wicked problems and clumsy solutions: the role of leadership. The new public leadership challenge.

[CR43] Hocquette A, Lambert C, Sinquin C, Peterolff L, Wagner Z, Bonny SP, Lebert A, Hocquette JF (2015). Educated consumers don’t believe artificial meat is the solution to the problems with the meat industry. Journal of Integrative Agriculture.

[CR44] Holmes T, Blackmore E, Hawkins R, Wakeford T (2012). The Common cause Handbook: a guide to values and frames for campaigners, Community Organisers, civil servants, fundraisers, educators, social entrepreneurs, activists, funders, politicians, and everyone in between.

[CR45] Hunter LM, Rinner L (2004). The association between environmental perspective and knowledge and concern with species diversity. Society and Natural Resources.

[CR46] IPCC (Intergovernmental Panel on Climate Change). 2019. Summary for Policymakers. In *Climate Change and Land: an IPCC special report on climate change, desertification, land degradation, sustainable land management, food security, and greenhouse gas fluxes in terrestrial ecosystems* (pp.1–35). IPCC.

[CR47] Johnson KA, Hill ED, Cohen AB (2011). Integrating the study of culture and religion: toward a psychology of worldview. Social and Personality Psychology Compass.

[CR48] Jones, B. A., D. Grace, R. Kock, S. Alonso, J. Rushton, M. Y. Said, and D. McKeever, 2013. Florence Mutua, Jarrah Young, John McDermott, and Dirk Udo Pfeiffer. Zoonosis emergence linked to agricultural intensification and environmental change. *Proceedings of the National Academy of Sciences, 110*(21), pp.8399–8404. 10.1073/pnas.1208059110 PMC3666729 23671097

[CR49] Judge M, Wilson MS (2019). A dual-process motivational model of attitudes towards vegetarians and vegans. European Journal of Social Psychology.

[CR50] Klain, S. C., P. Olmsted, K. M. Chan, and T. Satterfield. 2017. Relational values resonate broadly and differently than intrinsic or instrumental values, or the New Ecological Paradigm. *PloS one, 12*(8), p.e0183962. 10.1371/journal.pone.0183962 PMC5576695 28854227

[CR51] Kortenkamp KV, Moore CF (2001). Ecocentrism and anthropocentrism: Moral reasoning about ecological commons dilemmas. Journal of Environmental Psychology.

[CR52] Kubberød E, Ueland Ø, Rødbotten M, Westad F, Risvik E (2002). Gender specific preferences and attitudes towards meat. Food Quality and Preference.

[CR53] Laininen E, Cook JW (2019). Transforming our worldview towards a sustainable future. Sustainability, Human Well-Being, and the future of education.

[CR54] Lang, T., and M. Heasman. 2015. *Food wars: the global battle for mouths, minds and markets*. Routledge.

[CR57] Lensvelt EJ, Steenbekkers LPA (2014). Exploring consumer acceptance of entomophagy: a survey and experiment in Australia and the Netherlands. Ecology of food and nutrition.

[CR58] Makkar HPS (2018). Feed demand landscape and implications of food-not feed strategy for food security and climate change. Animal.

[CR59] Mata, J., P. Kadel, R. Frank, and B. Schüz. 2022. Education-and income-related differences in processed meat consumption across Europe: the role of food-related attitudes. Appetite, p.106417. 10.1016/j.appet.2022.106417 36521648

[CR60] Menozzi D, Sogari G, Veneziani M, Simoni E, Mora C (2017). Eating novel foods: an application of the theory of Planned Behaviour to predict the consumption of an insect-based product. Food quality and preference.

[CR61] NFS (National Food Strategy). 2020. *Your future, your food. Youth Consultation for the National Food Strategy* [Online]. [Accessed 10 January 2023]. Available from: https://www.nationalfoodstrategy.org/the-report/.

[CR62] NFS (National Food Strategy). 2021. *National Food Strategy Independent Review – The Plan* [Online]. [Accessed 10 January 2023]. Available from: https://www.nationalfoodstrategy.org/the-report/.

[CR63] NFU (National Farmers’ Union). 2019. *Achieving Net Zero, Farming’s 2040 goal* [Online]. [Accessed 10 January 2023]. Available from: https://www.nfuonline.com/nfu-online/business/regulation/achieving-net-zero-farmings-2040-goal.

[CR64] OECD (Organisation for Economic Co-operation and Development). 2021. *OECD-FAO Agricultural Outlook. Meats – 1992–2028* [Online]. [Accessed 10 January 2023]. Available from: https://stats.oecd.org/index.aspx?queryid=76854.

[CR65] Page, C., and B. Witt. 2022. A Leap of Faith: Regenerative Agriculture as a Contested Worldview Rather Than as a Practice Change Issue. *Sustainability, 14*(22), p.14803.

[CR66] Plumecocq, G., T. Debril, M. Duru, M. B. Magrini, J. P. Sarthou, and O. Therond. 2018. The plurality of values in sustainable agriculture models. Ecology and Society, *23*(1).

[CR67] Poore J, Nemecek T (2018). Reducing food’s environmental impacts through producers and consumers. Science.

[CR68] Prättälä R, Paalanen L, Grinberga D, Helasoja V, Kasmel A, Petkeviciene J (2007). Gender differences in the consumption of meat, fruit and vegetables are similar in Finland and the baltic countries. European Journal of Public Health.

[CR69] Rajasekaran, B. 1993. *A framework for incorporating indigenous knowledge systems into agricultural research and extension organizations for sustainable agricultural development in India* Doctoral dissertation, Iowa State University.

[CR70] Ramos-Castillo, A., and K. Galloway-McLean. 2012. Climate change mitigation with local communities and indigenous peoples: Practices, lessons learned and prospects. In *Proceedings of the international expert workshop climate change Mitigation with Local Communities and Indigenous Peoples*.

[CR71] Raygorodetsky, G. 2011. *Why Traditional Knowledge Holds the Key to Climate Change*. [Online]. [Accessed 10 January 2023]. Available from: https://unu.edu/publications/articles/why-traditional-knowledge-holds-the-key-to-climate-change.html.

[CR72] Rimal AP (2002). Factors affecting meat preferences among american consumers. Family Economics and Nutrition Review.

[CR73] Robinson JG (2011). Ethical pluralism, pragmatism, and sustainability in conservation practice. Biological Conservation.

[CR74] Rokeach M (2008). Understanding human values.

[CR75] Rosenfeld, D. L. 2020. Gender differences in vegetarian identity: how men and women construe meatless dieting. Food Quality and Preference, *81*.

[CR76] Rothgerber, H. 2013. Real men don’t eat (vegetable) quiche: Masculinity and the justification of meat consumption. *Psychology of Men & Masculinity, 14*(4), p.363.

[CR78] Ruby MB (2012). Vegetarianism. A blossoming field of study. Appetite.

[CR77] Ruby MB, Heine SJ (2011). Meat, morals, and masculinity. Appetite.

[CR79] Schösler H, de Boer J, Boersema JJ, Aiking H (2015). Meat and masculinity among young chinese, turkish and dutch adults in the Netherlands. Appetite.

[CR80] Schulz C, Martin-Ortega J (2018). Quantifying relational values—why not?. Current opinion in environmental sustainability.

[CR81] Schwartz, S. H. 2012. An overview of the Schwartz theory of basic values. Online readings in Psychology and Culture, *2*(1).

[CR82] Slade P (2018). If you build it, will they eat it? Consumer preferences for plant-based and cultured meat burgers. Appetite.

[CR83] Thompson M, Ellis R, Wildavsky A (1990). Cultural Theory.

[CR84] Van den Ban, A. W., S. V. N. Rao, D. V. Rangnekar, and K. Ranganathan. 1995. Indigenous technical knowledge and livestock. In *Handbook for straw feeding systems: principles and applications with emphasis on indian livestock production*, 119–128. Indo-Dutch Project on Bioconversion of Crop Residues.

[CR85] Verbeke W, Marcu A, Rutsaert P, Gaspar R, Seibt B, Fletcher D, Barnett J (2015). Would you eat cultured meat? Consumers’ reactions and attitude formation in Belgium, Portugal and the United Kingdom. Meat science.

[CR86] Vranken L, Avermaete T, Petalios D, Mathijs E (2014). Curbing global meat consumption: emerging evidence of a second nutrition transition. Environmental Science & Policy.

[CR87] Wardropper, C. B., A. S. Mase, J. Qiu, P. Kohl, E. G. Booth, and A. R. Rissman. 2020. Ecological worldview, agricultural or natural resource-based activities, and geography affect perceived importance of ecosystem services. *Landscape and Urban Planning, 197*, p.103768.

[CR88] Weiss R (1994). Learning from strangers: the art and method of qualitative interview studies.

[CR89] Wellesley, L., A. Froggatt, and C. Happer. 2015. *Changing climate, changing diets: pathways to lower meat consumption*. Chatham House, the Royal Institute of International Affairs.

[CR90] Wilks, M., and C. J. Phillips. 2017. Attitudes to in vitro meat: A survey of potential consumers in the United States. *PloS one, 12*(2). 10.1371/journal.pone.0171904 PMC5312878 28207878

[CR91] Willett W, Rockström J, Loken B, Springmann M, Lang T, Vermeulen S, Garnett T, Tilman D, DeClerck F, Wood A, Jonell M (2019). Food in the Anthropocene: the EAT–Lancet Commission on healthy diets from sustainable food systems. The Lancet.

[CR92] Williams RM (1968). Values. International Encyclopaedia of the Social Sciences.

[CR93] Zaremba, H., M. Elias, A. Rietveld, and N. Bergamini. 2021. Toward a feminist agroecology. *Sustainability, 13*(20), p.11244.

[CR94] Zurek M, Ingram J, Sanderson Bellamy A, Goold C, Lyon C, Alexander P, Barnes A, Bebber DP, Breeze TD, Bruce A, Collins LM (2022). Food system resilience: concepts, issues, and challenges. Annual Review of Environment and Resources.

